# Printed Crack Detection Sensors for SHM Based on Direct Ink Write Additive Manufacturing

**DOI:** 10.3390/ma19050870

**Published:** 2026-02-26

**Authors:** Artur Kurnyta, Klaudia Wrąbel, Marta Baran, Andrzej Leski

**Affiliations:** 1 Airworthiness Division, Air Force Institute of Technology, ul. Ksiecia Boleslawa 6, 01-494 Warsaw, Poland; 2 Institute of Aviation-Łukasiewicz Research Network, al. Krakowska 110/114, 02-256 Warsaw, Poland; 3 Belgian Nuclear Research Centre (SCK CEN), 2400 Mol, Belgium; 4 Institute of Mechanics, Materials and Civil Engineering, Université Catholique de Louvain (UC Louvain), 1348 Louvain-la-Neuve, Belgium; 5 Faculty of Mechanical Engineering, Military University of Technology, ul. gen. Sylwestra Kaliskiego 2, 00-908 Warsaw, Poland

**Keywords:** crack gauge, fatigue crack detection, printed sensor, structural health monitoring (SHM), fatigue crack growth (FCG), direct ink write (DIW), additive manufacturing, structural integrity

## Abstract

The following paper aims to provide the results of an innovative structural crack detection technique using printed adaptive sensors. They were manufactured using conductive ink with silver microparticles and polymer insulators. The technique leveraged the unique properties of Direct Ink Write additive manufacturing combined with domain knowledge in the field of technical condition monitoring. The goal was to achieve high sensitivity and precision in detecting fatigue-crack-induced changes in structural components. The sensors’ fabrication repeatability, output stability, and crack detection capabilities were investigated. Based on preliminary measurements of the sensors’ output characteristics, the analyzed data showed that a tolerance in the range of 5% can be obtained for batch production. Damage size estimation using this new crack gauge during a fatigue crack growth test was high compared to the reference, with less than 1 mm precision over 30 mm of crack length. Throughout the fatigue test of up to 1.5 million cycles, all CCPSs remained fully functional, with no failure-related changes in their output signal patterns. The proposed sensor has proven its reliability for the detection of fatigue cracks and propagation monitoring and is a good alternative to other SHM technologies for this purpose.

## 1. Introduction

Damage detection and monitoring of constructions is an essential part of the operational service life of various technical facilities. Facing the increased use of modern design methodologies and the utilization of new materials, periodic inspections alone are increasingly insufficient to ensure complete information concerning an object’s state with a high level of safety. A visibly growing trend of supplementing traditional non-destructive testing (NDT) techniques, such as eddy current testing, ultrasound, radiography, and thermography [1], with new real-time approaches based on structural health monitoring (SHM) concepts [2,3,4] can be seen. This is dictated by the need to improve operational safety, shorten the time required for technical maintenance, and automate the information analysis process, which also brings economic benefits [5].

The following work presents a new crack detection technique that utilizes printed, adaptive sensors fabricated with the use of conductive ink with silver micro-particles and polymer insulators. This advanced sensor technology is designed to detect and monitor early-stage damage, offering real-time diagnostics to enhance structural integrity and reliability for a wide range of applications, including in fields such as civil engineering (bridges, towers) and aerospace (fixed- and rotary-wing aircraft) and in industrial infrastructures. The delivered method combines additive manufacturing and material/mechanical engineering and measurement techniques with domain knowledge in the field of technical condition monitoring. Our motivation was to achieve high sensitivity and precision in detecting fatigue-crack-induced changes in structural components. By the customization of the sensors’ geometry, shape, and output characteristics, this damage detection technique can provide real-time monitoring and early warning capabilities, reducing or even minimizing the risk of catastrophic failures.

The Customized Crack Propagation Sensor (CCPS) is a new type of crack gauge specifically designed to detect surface fatigue cracks with the required accuracy and determine the size of the damage with a customized resolution [6]. This makes the sensor particularly effective for monitoring critical areas prone to crack propagation, both for new and aged structures. Compared to the commercially available sensors of that type, CCPSs can be tailored in shape to fit various structural geometries, ensuring better integration and coverage with a host component.

The presented technique expands the range of damage detection methods for SHM, where sensors/transducers and data acquisition have become an integrated part of technical objects. Various methods have been under evaluation, including passive and active piezoelectric transducers [7,8,9,10], eddy current testing [11], or strain-based techniques [12,13,14]. There are also a few examples of successful flight-proven system designs of SHM [15,16].

Crack detection methods more comparable to those investigated within the following paper, presenting an enhanced scope to that previously reported in [17,18], were also developed and tested by several research teams. Most of those methods are based on new designs of capacitive and resistive sensors. They are made of materials such as allotropic varieties of carbon (nanotubes, graphite, graphene) or metallic micro- and nanoparticles (mainly silver and copper) in the form of additives, pastes, or inks to be applied on the host structure.

In the papers [19,20], an ICM (Intelligent Coating Monitoring) sensor was described. A sensitive layer was made from a water–ethanol suspension with copper nanoparticles or a copper-plated layer created using pulsed-arc ion polarization. The single conductive strand around the hole in the specimen was intended to inform about developing damage when the conductivity is lost due to strand breakage. A similar approach was delivered in [21] but with a strand developed manually from a silver conductive paste. A dedicated electronic circuit was designed to turn on a LED diode and signal a break in the sensor’s electrical continuity. In [22,23], another approach with a single-strand crack detection sensor was proposed and tested on an aluminum alloy plate, as well as in the study of the quasi-static bending of an Airbus A320 aircraft element with existing damage. The insulating layer for the electrically conductive path was an epoxy primer used as anti-corrosion protection for aircraft structures. Within this research, the additional influence of crack closure and reconnection of a broken strand was investigated for the sample in the unloaded state. The above methods have a common capability limited only to detecting cracks but without the feature of being able to monitor their propagation and size estimation.

In [24,25], examples of crack detection sensors, made of carbon nanotubes as an electrically conductive layer in the form of foils, were presented. Based on the conducted tests, it was proven that the proposed sensors are able to perform continuous and real-time crack monitoring by measuring changes in electrical resistance. No false positives were observed, and an additional test to determine the methods’ sensitivity to reinitializing crack propagation from a drilled hole was also demonstrated effectively. However, during the fatigue crack propagation in the host structure, the observation of the crack tip was limited. The reason were mainly due to the use of opaque materials, the sensor being in the form of a foil or adhesives used for transducer integration with the host structure.

Another innovative crack detection method, again with a carbon-nanotube-based sensitive layer, was proposed in [26,27]. A soft elastomeric capacitor (SEC) was designed to detect large strain changes, which affect the sensor’s electrical capacitance. These sensors demonstrated sensitivity to cracks propagating in a metal sample but with a significant delay in response. Unlike the methods mentioned above, this sensor does not crack along with the structure. It is flexible, bonded to the tested structure, and deforms as the crack grows, and the change in capacitance serves as a diagnostic signal. During the tests, the sensor readings were correlated with the results of visual inspection. A significant and sudden change in capacitance was observed when the crack propagated under the sensor for a distance of 27 mm.

In view of the increasingly widespread use of composite structures, another group of methods for detecting damage in these materials is related to the natural electrical conductivity of carbon-fiber-reinforced polymers (CPRF) composites [27,28,29,30]. These methods are sensitive to fiber and composite cracks, detecting impact damage, interlaminar delamination, and cracks propagating from rivets. In [29], it was shown that a four-wire configuration demonstrated better sensitivity to resistance changes due to damage, instead of using the classic two-wire connection. However, these methods again provide more qualitative than quantitative information for estimating damage size. Therefore, the transition from resistance change to damage size may require an individual approach to the calibration process.

In summary, the methods presented in the previous section have demonstrated overall effectiveness in detecting damage, particularly cracks. However, limited visual verification of the crack tip location hinders accurate comparison with the actual extent of damage in the host structure. Additionally, it was observed that the relative resistance did not change linearly with crack progression, making it difficult to calibrate the sensor in units of crack length. Single-strand sensors provide only crack detection without monitoring propagation. CCPS design addresses these specific shortcomings. The use of transparent insulation and adhesives enables the assessment of crack tip location. A grid-shape-sensing layer provides enhanced interpretation of the sensor output signal and a clear correlation of voltage changes with the damage size.

The fabrication method of CCPS sensors is based on additive manufacturing, known from printed electronics. In general, printed sensors are ideal for large-scale sensing applications. Given their cost-effectiveness and adaptive form factor, they are an excellent fit for various sectors, including environmental, agricultural, medical and structural sensing [31]. In [32,33], development and fabrication methods, conductive and insulating materials, printing technologies and applications were discussed. A critical review of printed sensors fabricated on flexible platforms such as paper, plastic and textiles is delivered. The implementations for wearable applications in the biomedical, defense, food, and environmental industries are described. Reported printed devices such as TFTs, LEDs, sensors, displays, solar cells, RFID tags, batteries, energy harvesters and capacitors are the consequence of increased interest in the area of printed, flexible and hybrid electronic applications [34].

Examples of utilizing printed electronics manufacturing in new or refreshed applications are numerous. In [35], planar silkscreen-printed elasto-resistive sensors were described, combined with Electrical Impedance Tomography (EIT) signal processing. A capacitive polyimide-based force sensor with an elastomeric dielectric layer was proposed in [36], fabricated by screen printing and casting methods. Tests revealed stable readings in the presence of changes in relative humidity and temperature. In [37], a review of flexible strain sensors fabricated by various printing technologies is delivered. The key findings indicate that the operational parameters and durability of the sensor are highly dependent on the proper selection of conductive ink and substrate compatibility with different printing techniques. Printed sensors also present unique capabilities of combining different transducers within a single circuit with a low footprint. For example, in the work [38], the development and characterization of multifunctional, wireless, and battery-free biface sensor tags, which consist of temperature, humidity and strain sensors, are researched. It addresses the focus on sustainability by the use of uncoated paper and resource-efficient manufacturing techniques. The printed electronic’s Life Cycle Assessment (LCA) and its environmental impact were also investigated in [39].

Within the following paper, a dispensing, or so-called Direct Ink Write (DIW), manufacturing technique combined with foil casting was utilized for CCPS sensor fabrication. DIW is one of the prototyping methods where the manufactured design is easy to modify. Changes to layout and printing parameters can be made without using additional tooling [40,41,42]. Screen printing, on the other hand, uses the prefabricated screen on which the pattern is prepared for the intended application. Moreover, DIW can work with a wide range of materials by means of viscosity, which is not the case for, e.g., ink-jet printing [43,44,45].

However, printed electronic devices, regardless of the technique and materials used, still face challenges before they can be fully incorporated in the industry. Few performance, reliability and stability issues of printed circuits have been addressed in the literature. In [46], substrate materials were investigated for a flexible piezoresistive pressure sensor. Key material attributes were found to play an important role, like Young’s modulus, glass transition temperature, coefficient of thermal expansion (CTE), thermal conductivity or water absorption. Since the CCPS sensor grid is subjected to cyclic mechanical loading, the following paper delivers new findings in addition to the results reported in [47,48,49]. Those papers address the issue of printed circuit degradation and changes in electrical parameters due to cycling tensile and bending loading. In terms of the materials used and their applications, a relationship was identified between the geometry and porosity of the electrically conductive layer and the operational reliability of passive electronic components under cyclic mechanical loading. This is a reason for the importance of print quality evaluation and dimensional accuracy, as discussed in [50]. A correlation was also observed between electromechanical properties and the size and shape of the electrically conductive particles [51]. It was noted that the presence of a protective top layer significantly impacts the flexibility of printed traces with silver particles. A lack of output signal stability could also be a consequence of insufficient or environmentally degraded adhesion between the circuit conductive and insulating layers [52,53].

To conclude, the investigation in the following paper addresses the growing need for enhanced safety in construction and infrastructure maintenance. The solution combines cutting-edge material science with an intelligent, host-structure-integrated detection system. The sensor can be tailored to the host structure by means of shape and output characteristics, improving accuracy and decreasing inspection intervals due to real-time monitoring. This research activity represents a significant advancement in the field of SHM, offering a scalable, cost-effective solution to ensure the long-term durability and integrity monitoring of critical areas of structure or construction elements. Additionally, the uniqueness of this proposed sensor lies in the combination of cost-effective and rapid prototyping techniques, such as tape casting and DIW deposition, for the development of electronic devices. Indirectly, both operation and reading stability under cyclic loading were verified for those printed sensors. The test article was subjected to more than 1.5 million mechanical cycles, which also addresses the issue of fatigue-related degradation of the conductive layer and interlayer as well as bonding adhesion. Finally, we have shown that the CCPS has proven potential for reliable fatigue crack detection and propagation monitoring and is a good alternative to other SHM technologies for this purpose.

## 2. Materials and Methods

### 2.1. Materials

A 3 mm thick aluminum alloy 2024-T3 (Table 1) with a brushed finish was selected as a host material due to its relevance in aerospace component manufacturing (e.g., wing stringers, skin) [54]. The material was used to prepare a standardized specimen for a fatigue crack growth test. Before installation of sensors, the specimen surface was grinded with 400-grit sandpaper, chemically cleaned using CSM-3 Degreaser (Micro-Measurements-Division of Vishay Precision Group, Raleigh, NC, USA) and ATEPO Z-12 (Tenmex S.C., Łódź, Poland), followed by neutralization with ATEPO Z-03 (Tenmex S.C., Łódź, Poland). Dust-free swabs for cleaning surfaces were used to apply chemical agents.

For manufacturing the CCPS sensor, commercially available silver conductive paste DM-SIP-3170S (Dycotec Materials Ltd., Wiltshire, UK) was used to fabricate the active sensing layer. For the bottom as well as the top insulating layer, two-part epoxy 832C (MG Chemicals, Burlington, ON, Canada) was used. Side electrodes of the sensor were cut out from embossed, tin-plated copper foil adhesive tape 1345 (3M, Colorado City, TX, USA).

For the sensor installation to the host structure, cyanoacrylate adhesive M-Bond 200 together with a dedicated catalyst (Micro-Measurements-Division of Vishay Precision Group, Raleigh, NC, USA) was selected for its certified use in bonding strain gauges. Just before the application, surfaces of the host specimen (only left side from the notch) and the bottom of sensors were both manually treated with atmospheric-pressure plasma to enhance wettability and increase bonding strength. A PiezoBrush PZ3 device (Relyon Plasma GmbH, Regensburg, Germany) equipped with a Nearfield Module was utilized for that task.

### 2.2. CCPS Sensor Fabrication

**Figure 1:**
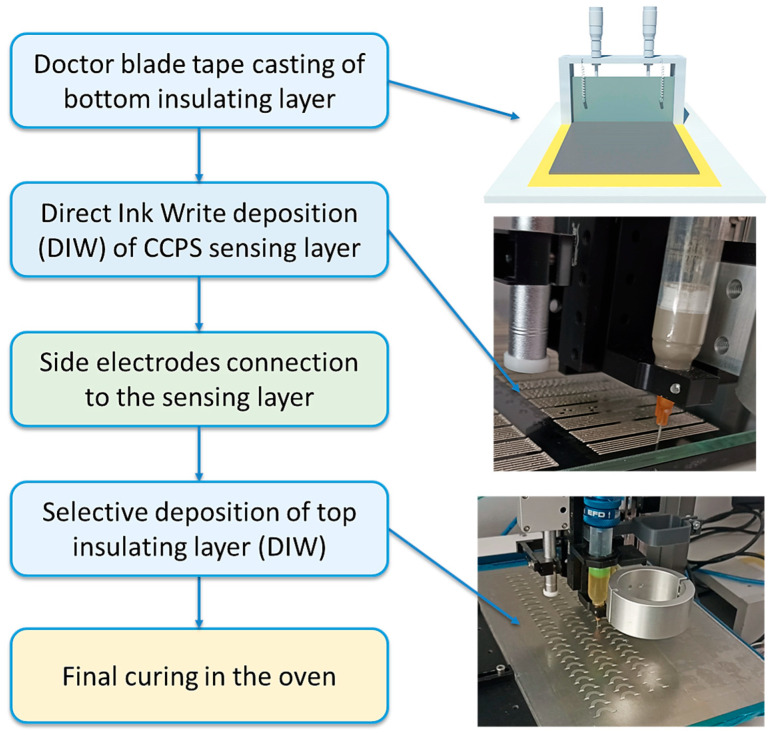
Main stages of CCPS sensor fabrication process.

The CCPS fabrication method is illustrated in Figure 1. First, the epoxy thin film was prepared to serve as a bottom insulating layer for a batch of sensors. It was manufactured by the tape casting method and doctor blading to achieve a desired thickness of approx. 60 µm. The coater MSK-AFA-III-HB and the film casting tool SeKTQ100 (MTI Corporation, Richmond, CA, USA) were used. Prior to application, 2-part epoxy resin 832C was manually mixed thoroughly with a spatula in a 2:1 ratio, as instructed by the manufacturer, and left for degassing for 30 min. After casting the film on a PE tape adhered to 10 mm aluminum alloy plate, it was placed in a heating oven for 2-stage curing at temperatures of 30 °C and 65 °C, both for 60 min. The final film thickness was verified afterwards by an Elcometer 456 eddy-current thickness gauge (Elcometer, Manchester, UK).

The sensing layer was selectively deposited on the cured epoxy film by the DIW method with DM-SIP-3170S silver conductive paste. The conductive paste, used with no prior modification, was poured into syringe with a nozzle and placed in the mount of a Nordson E3V series 3-axis table robot equipped with an Ultimus V series time-pressure dispenser device (Nordson EFD, Norwich, CT, USA). The sensing grid layout was prepared in the robot’s dedicated DispenseMotion Software (v. 2.36) to define the nozzle’s path over the epoxy substrate. Optimization of the deposition process parameters (nozzle diameter, nozzle height over substrate, applied pressure to syringe, nozzle movement speed) was performed in advanced, but its results are beyond the scope of the following paper. The final printing parameters were as follows: printing speed 20 mm/s, extrusion pressure 80 PSI, 10 mL syringe with 0.2 mm inner diameter nozzle. The deposited layout was dried in the oven for 20 min at 60 °C before placing the side electrodes. Another pass of a nozzle dispensing conductive paste was intended to firmly connect the electrodes to the sensing layer, followed by final curing of the layer in the oven for 20 min at a temperature of 100 °C. The printing speed was reduced to 10 mm/s at that stage.

For the top layer, the same method as for the sensing layer was used, producing a plane but geometrically fitted protective coating over the sensor using 832C epoxy. Again, this layer was cured in the heating oven at a temperature of 65 °C for 60 min. After cooling, sensors from the batch were cut to size using a laser plotter (LaserPro Spirit LS, GCC, New Taipei City, Taiwan) and detached from the PE tape technology plate.

### 2.3. Sensor Electrical Measurements

CCPS is a parametric, resistive-type sensor, and its prime parameter is equivalent resistance. A precision digital multimeter MetraHit 30M (Gossen Metrawatt Gmbh, Nürnberg, Germany) provided accurate resistance measurements of the sensors before and after installation on the host structure. Selected sensors from the batch were also evaluated to verify the repeatability of the output resistance characteristics. During the test, subsequent strands of the sensor’s electro-conductive grid were cut with a scalpel blade, and resistance measurements were taken each time to obtain the output characteristics.

**Figure 2:**
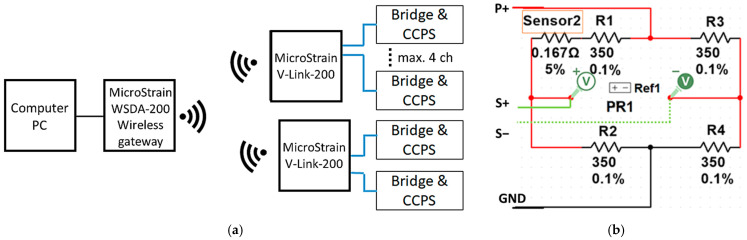
Block diagram of the acquisition systems: (**a**) main scheme, (**b**) detailed bridge-type sensor conditioning circuit for one measuring channel.

In the main part of the research, which was fatigue crack propagation in an aluminum alloy host specimen instrumented with CCPS, real-time measurements were collected. Multichannel sensor modules with bridge-type input were used to acquire data from the sensors (V-Link-200 sensor node, MicroStrain, Williston, VT, USA). For each measuring channel, the sensor was fitted in series in one arm of a Wheatstone Bridge composed of 350 Ω precise resistors (Elpod, Cracow, Poland). The application of such a measuring system allowed for the demonstration of the possibility of using the CCPS sensor in the same connection as for a common strain gauge [55] (Figure 2). For the detailed conditioning measuring circuit shown in Figure 2b, R1 ÷ R4 are the bridge complement resistors, *P*+ is a connection point of positive excitation voltage, GND stands for negative excitation, and *S*+ and *S*− are connections for the output signal from the circuit. V-Link-200 provides a wireless connection with the PC via the gateway WSDA-200-USB to acquire data remotely from the specimen.

The dedicated desktop software SensorConnect (v14.11.0) was used for node configuration, channel ranging and balancing, data visualization and exporting to a text file after completing the test.

### 2.4. CCPS and Host Structure Visual Inspection

Visual inspection with digital optical microscopy (VHX 6000, Keyence, Osaka, Japan) was utilized at several stages of the investigation. Selected manufactured CCPSs were verified as a quality check procedure for sensing layer continuity, strand waviness and grid-to-electrode connection. After installation of the crack sensors on the specimen, the microscope was used to measure distances between the mechanical notch and the location of the CCPS sensing layers. After completion of the fatigue test, VHX-6000 was used to investigate the specimen crack propagation course, fracture surface analysis, and broken edges of the CCPSs due to crack growth.

During the fatigue test, additional visual inspection of the crack tip and CCPS strands was conducted periodically with an EVO Cam II microscope (Vision Engineering Ltd., Woking, UK). However, images from this device were not marked and synchronized with the number of cycles applied to the M(T) specimen, although it was possible to reveal the CCPS sensing layer breakage compared to the crack tip in the specimen.

### 2.5. Fatigue Crack Growth Test Set-Up and Procedure

**Figure 3:**
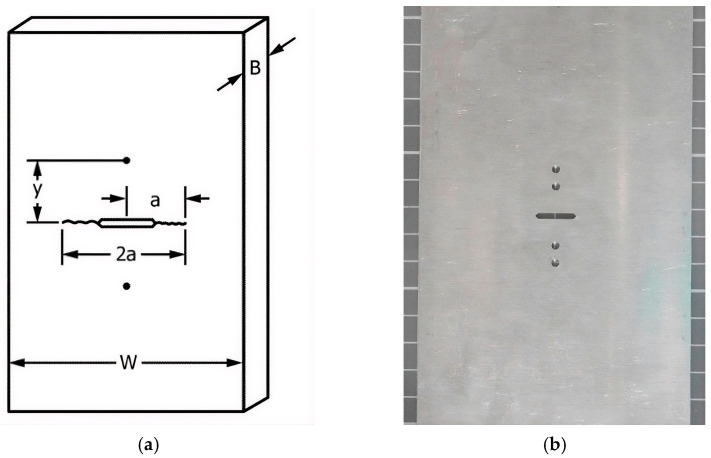

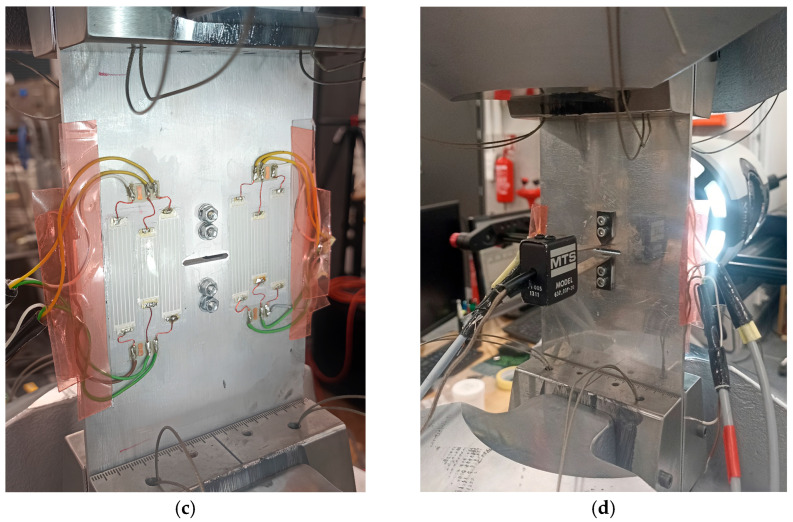
Standardized the M(T)-type specimen (**a**) schematic diagram with the parameters necessary to determine the crack length using the compliance method, (**b**) view of the specimen manufactured from 2024-T3 aluminum alloy, (**c**) front side of the specimen with CCPS mounted on the test stand, and (**d**) rear side of the specimen mounted on the test stand with COD.

The test stand for the fatigue test was conducted on MTS 810.23 servo-hydraulic materials tester (MTS-Systems, Eden Prairie, MN, USA). To perform the test and obtain fatigue crack growth (FCG) in a controlled and reproducible manner, the setup and execution were carried out based on the ASTM E647 standard [56]. A standardized M(T) (Middle Tension) specimen was chosen as a host structure for CCPS verification with the geometry shown in Figure 3 and Table 2. The M(T) specimen had a mechanical notch located in the center part, mimicking more “plane stress” or lower constraint conditions, and this better reflects the behavior of thin sheets [57].

Utilizing the standard procedure for FCG gave the benefit of using automatically calculated reference crack length estimation in the host structure by a Crack Opening Displacement (COD, 632.03F-30 from MTS-Systems, Eden Prairie, MN, USA) sensor, as shown in Figure 3d, based on the specimen compliance method [56]. The current crack length *a* in the specimen was determined for CCPS reference based on the material properties of 2024-T3 alloy (Table 1 and Table 2), the mechanical load amplitude *P*, and the established measurement base *y* for COD readings.

The FCG test involved a 2-stage process:

- Formation of a pre-crack;
- Constant-amplitude (CA) dynamic loading.

Loads and crack length measurements were performed during the fatigue test using the compliance method within the automated measurement procedure of the testing machine manufacturer: MTS 793.40 (MTS-Systems, Eden Prairie, MN, USA) (fatigue crack growth).

## 3. Results

The following part presents the results from the evaluation of the CCPS sensors. Firstly, selected sensors from the manufactured batch were verified for their output resistance characteristic repeatability. Secondly, a fatigue crack growth test was performed for the M(T) specimen with six CCPS sensors installed. Finally, post-test images and measurement results of visual inspection of the M(T) specimen are delivered. Both the sides and the fracture surfaces were investigated.

### 3.1. CCPS Batch Output Resistance Measurements

**Figure 4:**
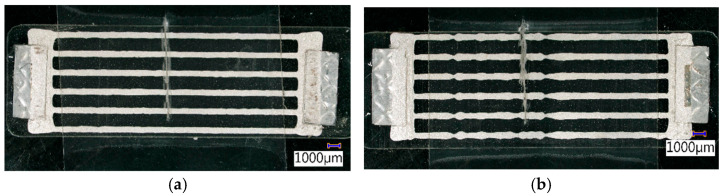
Examples of CCPS manufacturing quality variation, (**a**) satisfactory (CCPS_P1), (**b**) unsatisfactory (CCPS_B4) with a scalpel cut.

Before the investigation of the CCPSs during the FCG test, five sensors from the same batch were selected for the static resistance measurements. The goal was to obtain the full sensor output characteristics by gradually cutting the sensing layer strands one-by-one. The resistance measurements were collected for each step with a precise multimeter. Sensors with varying levels of manufacturing quality were deliberately selected for the test (Figure 4). The most common factors contributing to a lower quality rating were local narrowing of the conductive grid strands or imperfect connections between the side electrodes and the deposited conductive material (Figure 4b).

**Figure 5:**
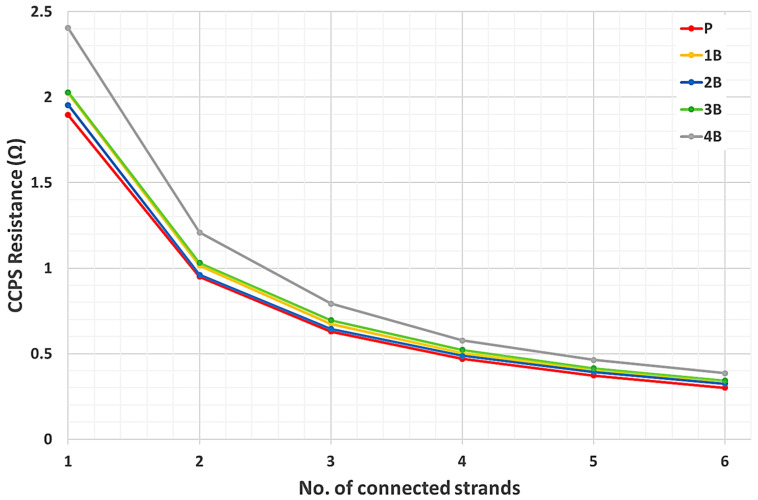
Resistance output characteristic from CCPS sensors.

The resistance measurement results for five selected sensors are shown in Table 3. Because two wire connections were used, short connection readings for the digital multimeter leads were obtained, and their values were subtracted from the sensor measurements. The resistance measurement results are also presented in a graphical form in Figure 5 to visualize the fabrication repeatability of the selected sensors within the production batch.

As the CCPS sensing grid layout presented in the following paper is a simple shape, its output characteristic should be equivalent to the parallel connection of resistors with the same *R* value. This assumption enables the analysis of the theoretical characteristics of the CCPS sensors taken for evaluation. The theoretical characteristic for each sensor was calculated in two ways, taking as a base value the CCPS resistance measured for: (a) a fully connected grid and (b) only the last strand connected (for the case of an increasing number of connected strands).

For the first case, which corresponds to natural sensor operation mode and resistance change due to a decreasing number of connected strands, the equation for the theoretical characteristic is as follows:

(1) Rn−1=Rn·(nn−1)

where *n* indicates the successively decreasing (from *n* = 6 to *n* = 1) number of connected strands within the sensing grid, and *R_n_* and *R_n_*_−1_ are the equivalent resistances for an indicated number of connected sensor strands. The known base resistance value for further calculations is *R*_6_ for a fully connected grid.

For the second case, calculations were performed by taking the resistance value for a single uninterrupted sensor strand as the base value. The following equation was used:

(2) $$R_{n+1}=R_{1}/\left(n+1\right)$$

where *n* indicates the successively increasing (from *n* = 1 to *n* = 6) number of connected strands within the sensing grid. *R_n_*_+1_ is the equivalent resistance for an indicated number of connected sensor strands. The known base resistance value for further calculations is *R*_1_ for a single-strand grid.

**Figure 6:**
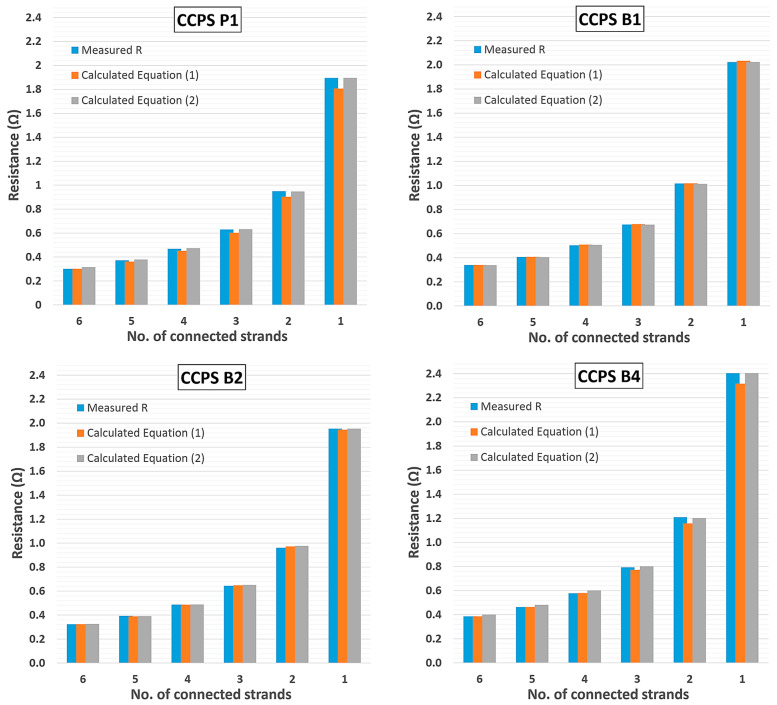
Comparison of the measured and theoretical resistance characteristic for selected CCPS.

In Table 4, the calculation results based on Equations (1) and (2) are shown. The theoretical resistance characteristics revealed differences in the resistance value for a certain number of connected strands for several sensors. Figure 6 illustrates a comparison of the measured and theoretical resistances for selected CCPS sensors due to sensing grid breakage.

### 3.2. Fatigue Crack Growth Test Execution

The following part presents the execution and results of an FCG test for the M(T) specimen with permanently integrated CCPS sensors. The goal was to verify the ability of the transducers to detect and track crack propagation within the host structure. In total, six CCPSs were installed on the specimen (Figure 3c), three on each side of the mechanical notch, one-by-one. Detailed locations for each of the sensing grids relative to the notch are delivered in the next section. It should be noted that only the middle CCPS on each side of the notch was geometrically identical, as with the sensors evaluated in the previous section. The others were manufactured with 50% longer strands within the sensing grid but with the same strand number and separation (1.5 mm). This demonstrates sensor shape customization.

**Figure 7:**
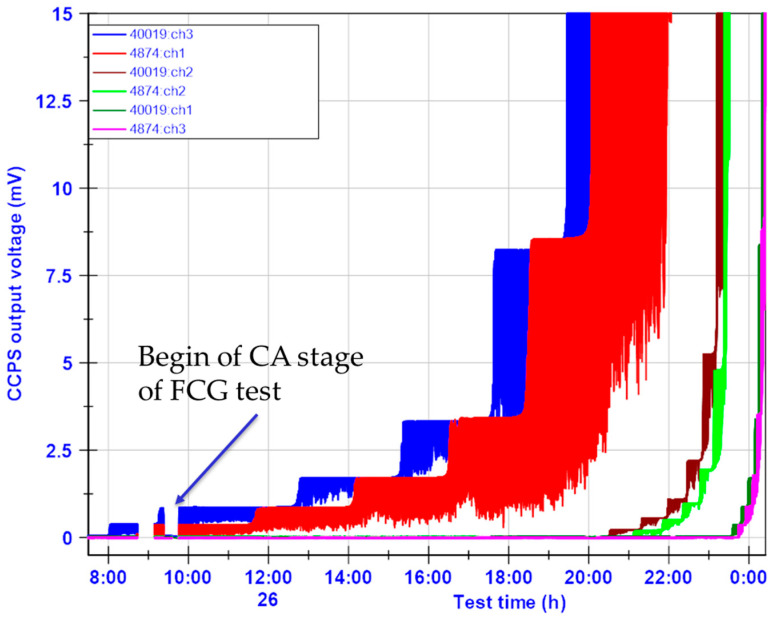
Unprocessed time series data from CCPS sensors during the FCG test on the M(T) specimen.

Unprocessed acquired data for all CCPSs are shown in Figure 7 as a time series. The measuring channels’ name convention is as follows:

- The CCPS installed on the left from the M(T) mechanical notch begins with the number 40019, and this continues with the sensor channel number, from left to right. As a consequence, 40019:ch3 is the closest sensor to the notch left tip;
- The CCPS installed on the right from the M(T) mechanical notch begins with the number 4874, and this continues with the sensor channel number, from left to right. As a consequence, 4874:ch1 is the closest to the notch right tip.

**Figure 8:**
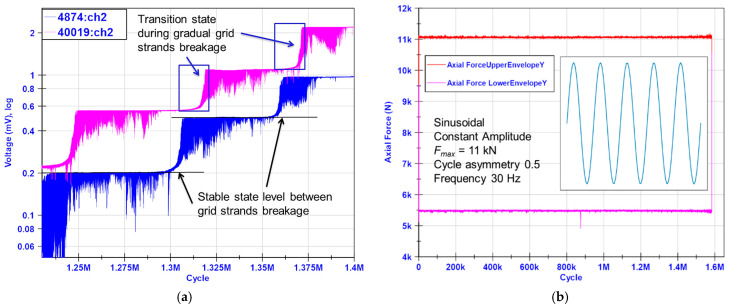
(**a**) Interpretation of data acquired from CCPS, (**b**) fatigue crack growth test force representation applied to M(T) specimen during CA stage.

Figure 7 presents the acquired data from both the pre-crack and CA stage of the test. Due to a lack of logged data from the test stand parameters (*P*—applied force, and *a*—COD-based crack length) for the pre-crack stage, further results and analysis are based on the constant-amplitude stage only (Figure 8b). However, it can be noticed that two strands for the left 40019:ch3 and one for the right 4874:ch1 CCPS sensors were broken within the pre-crack stage.

Figure 8a delivers an explanation for how to interpret the data from the CCPS for crack detection and monitoring, where the stable states’ output voltage periodically shifts up due to sensing grid strand breakage with the host structure. The breakage is manifested with a so-called transition state, in which the conductive strand is gradually torn apart by the propagating crack tip in the specimen. This causes a monotonic increase in the maximum output voltage level until it achieves a new stable state, when the conductive strand is fully broken.

The crack in the sample changes its opening during each cycle due to the force applied. This can also be observed in the changes in the CCPS measured voltage signal for a broken strand. For this reason, the useful diagnostic component of the output signal is a local maximum voltage when the crack is fully opened. To facilitate CCPS data interpretation and increase the readability of the results, the raw signals were recalculated to obtain only the upper envelope of the signals from all sensors.

**Figure 9:**
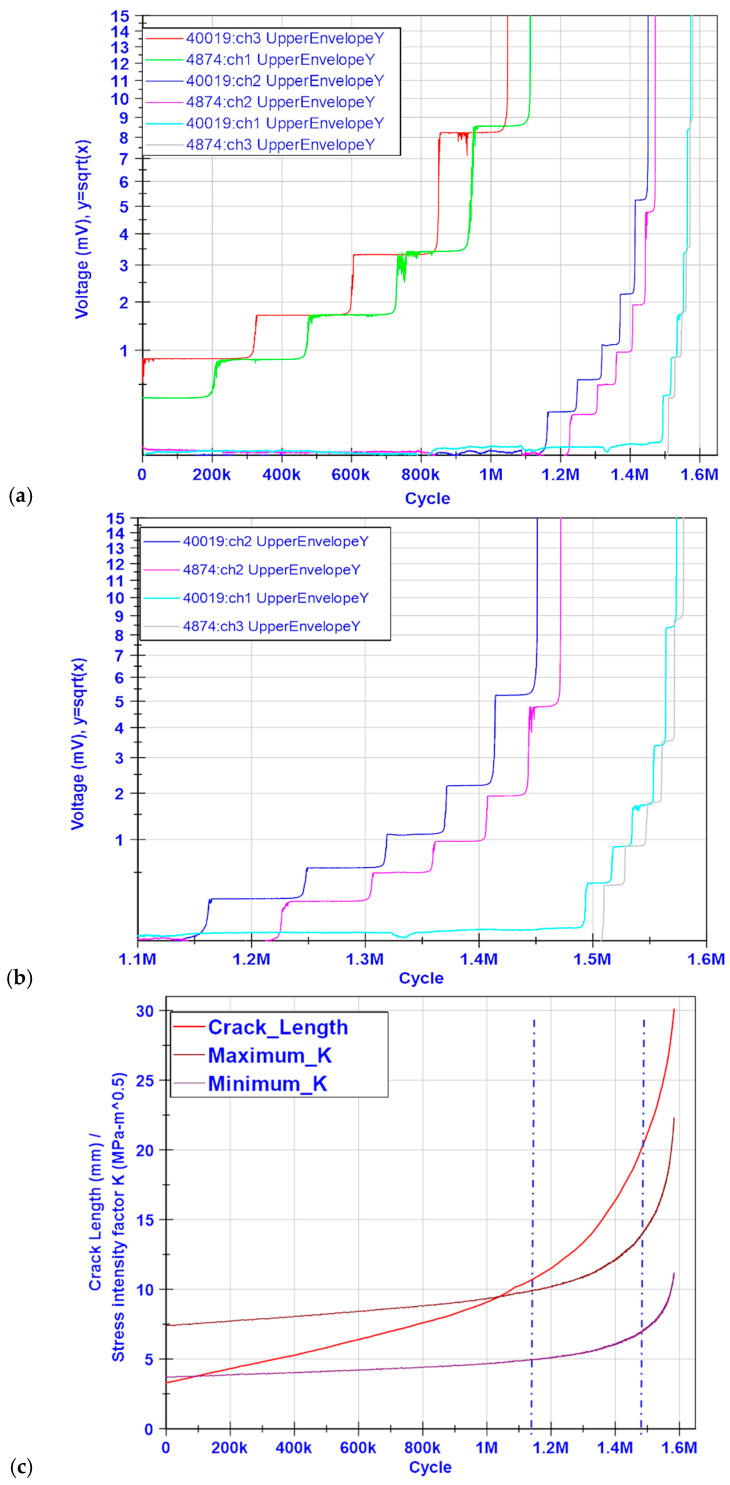
Processed data from the FCG test (**a**) for all CCPSs, (**b**) close-up view for sensors with a higher crack growth rate, (**c**) crack length based on COD readings and stress intensity factor during test.

For each pseudo-step-wise CCPS characteristic, the increase in the crack length in the specimen can be clearly distinguished (Figure 9a,b). Transition states prove that the sensing grid is torn apart successively, not in a single cycle. The stable states show a strong sideways trend, with no noticeable alterations. The number of signal level shifts corresponds with the number of strands within the sensing layer for each CCPS. Figure 9c illustrates a crack length estimated from the COD sensor mounted on the M(T) specimen, as well as the calculated values of the minimum and maximum stress intensity factor during cycling.

The stress intensity factor (*K*) is a critical parameter in linear–elastic fracture mechanics (LEFM) that quantifies the severity of the stress state at a crack tip [57]. Its calculation is based on the load (applied force), crack size, and structural geometry of a test article. It is the core parameter that allows for estimating how fast a crack is growing under fatigue (Paris’ Law) and predicting when it will fail. K, as a function of the crack size, represents the stress field at the crack tip. The blue vertical dashed lines in Figure 9c indicate the working area of the CCPS sensors in relation to the number of fatigue cycles. The pair of CCPSs closest to the notch (40019:ch3 and 4874:ch1) operated at a relatively low crack growth rate with a linear trend. However, the last pair of CCPSs worked in a region where both the crack length increase and the stress intensity factor became non-linear with the number of cycles. This means higher stress values in the crack tip area, typical for the final phase of a fatigue test, just before the complete breakage of the host specimen.

Data from the CCPS and COD were synchronized as a function of fatigue cycles. Visual determination of the CCPS sensing grid’s position relative to the notch enabled a comparison of the current specimen crack length readings from both sources. It was assumed that complete strand breakage and reaching a new stable output signal level was equivalent to obtaining a specific crack length at the indicated number of cycles. A comparison of the crack length with calculated differences is shown in Table 5.

**Figure 10:**
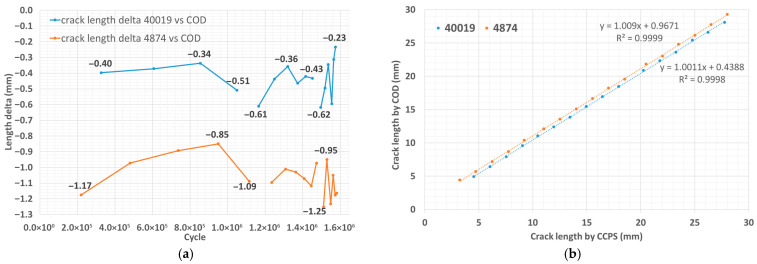
Specimen crack length comparison from CCPS sensors and COD: (**a**) illustrated differences (delta), (**b**) trend line comparison for left (40019) and right side (4874) of the M(T) specimen.

The results summarized in Table 5 are illustrated graphically in Figure 10a to show the differences for the CCPS and COD crack length values. Figure 10b shows that despite the differences in the crack length readings from both sources, there is a strong linear relationship between damage size increments.

### 3.3. CCPS and Host Structure Visual Inspection After FCG

**Figure 11:**
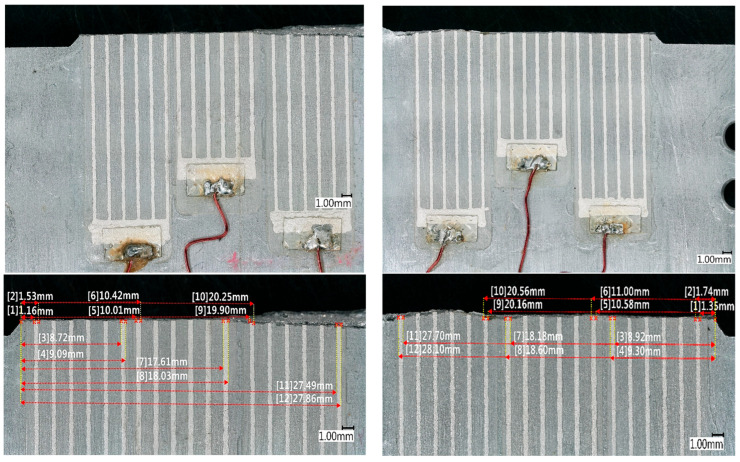
Microscopic images of the specimen after FCG test completion, with marked distances of the CCPS sensing grid from the notch.

The locations of the CCPS sensors relative to the mechanical notch of the M(T) specimen were measured with a microscope before and after the FCG test. The post-test visual inspection findings are delivered in Figure 11. The top images show the crack edges of the host structure and the CCPS sensors in the area of damage. The bottom images illustrate the true locations of the sensing grids along the crack propagation path.

The post-test microscope images of the crack edges reveal that both the host structure and CCPS damage lines are smooth and equal in the area of the inner and middle CCPS sensors. In the area of the bonded outer sensors, changes in crack line and shape are noticed, which correspond to damage progression within a higher stress intensity factor value. The crack in this area began to develop much faster (Figure 9c) and in a less controlled manner. There were slight lacerations of the edges on both the sample and the sensor.

**Figure 12:**
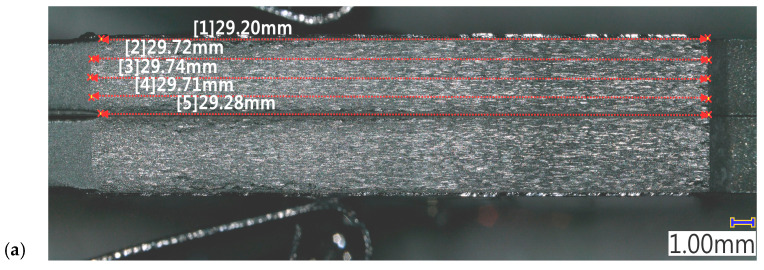

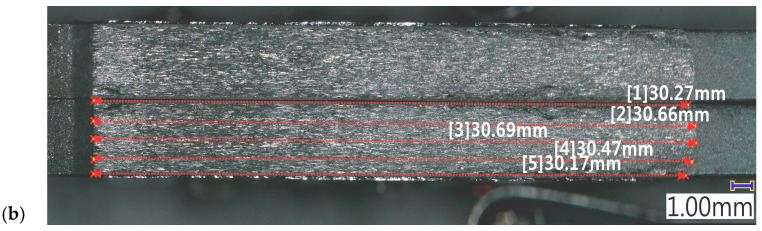
Microscopic images of crack surface with true length verification: (**a**) left side of M(T) specimen, (**b**) right side of M(T) specimen.

Fatigue fracture surface analysis is delivered in Figure 12. This revealed differences in the actual crack length for both sides of the M(T) specimen of almost 1 mm, as well as between the middle part of the cross-section and the outer edges, ranging from 0.40 ÷ 0.54 mm. The readings from the COD crack length estimation give only one output number and do not distinguish the direction of crack propagation. It is assumed within the standard that the crack size is equal in both directions and that it has almost a flat front in the cross-section. This could be the main source of discrepancies between the crack length obtained from the CCPS and the COD reference. Despite the use of a standardized specimen and a reference crack length measurement in accordance with the guidelines, the above results indicate the possibility of slight deviations in the host structure indications.

**Figure 13:**
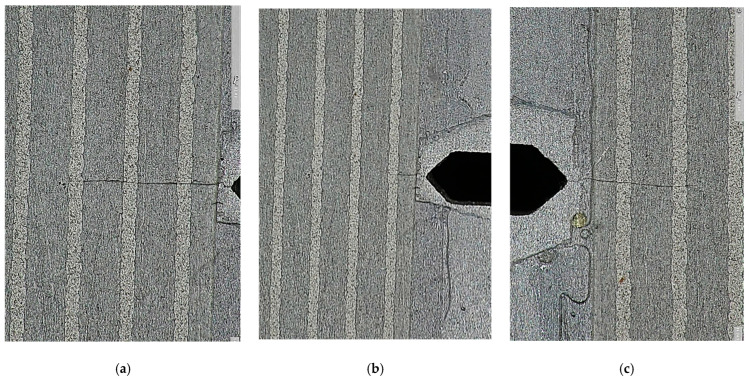
Microscopic images of crack propagation in the M(T) specimen during the test (**a**) in the middle of the third strand on the left side, (**b**) first strand on the left side almost fully broken, (**c**) crack just crossing the second strand on the right side.

Due to the above, photos of the crack were taken periodically to ensure even CCPS sensing layer tearing with the crack tip location in the host structure. The microscopic images in Figure 13 confirm that the crack tip of propagating damage in the specimen did not outstrip the breakage of the CCPS strand. Additionally, it was also affirmed that the conductive strand was torn successively and not only when damage in the host structure was visible beyond the grid.

## 4. Discussion

The presented research activities evaluate CCPS sensors as a crack detection solution for SHM. Firstly, the sensors’ fabrication repeatability was assessed for selected sensors from one production batch. The CCPS sensing layer was cut with a blade, and resistance measurements were collected. Output resistance characteristics as a function of the number of connected strands were obtained and are presented graphically in Figure 5. Despite the selection of five sensors with a different level of printed grid quality, the resistance values for an equal number of connected strands were within a narrow tolerance. For a fully connected grid with six strands, the differences in measurement were mostly below 0.04 Ω, while for the single strand the difference was 0.13 Ω. The sensor CCPS B4 showed a larger shift, but the quality of the grid produced was classified as unsatisfactory. However, this sensor was deliberately selected for a study to assess the potential spread of data within the manufacturing batch. The resistance change due to the CCPS strand cuts was almost equal for most of the sensors.

The coefficient of variation (CV) was calculated based on data from all sensors with the following equation:

(3) CV = Standard deviation/mean ×100%

which can express the spread of data. The statistics for the data are shown in Table 6. It can be noticed that the absolute repeatability for the tested CCPS sensing layer is below 9%. Excluding the CCPS B4 sensor, it became significantly better, reaching almost the 5% tolerance range. This observation is valid for the tested samples presented in the paper. In future work, a statistically significant number of samples will be used to perform rigorous validation of this finding.

The CCPS sensing layers presented in this paper are composed of strands with an equal geometrical shape. For this reason, calculations of the theoretical resistance characteristic were performed based on Equations (1) and (2), and these are presented in Table 4 and Figure 6. Very good correspondence to the measurement data was revealed, maintaining an alteration range of 0.5 ÷ 5% for all CCPSs evaluated from the batch. The differences are not clearly correlated with the method of calculating the theoretical value of the resistance with Equation (1) or Equation (2).

However, as fabrication repeatability is a concern, the findings from this part of the CCPS investigation are similar or even enhanced compared to the literature. In [58], tolerances better than 10% for custom embedded resistors were obtained, but an advanced Physical Vapor Deposition (PVD) method, additional shadow masks, and precision alignment were utilized. In [59], similar tolerance was reported for screen-printed resistors with oxide–metal coatings. To obtain a lower tolerance, trimming is a common activity. However, for CCPS application, the resistance deviation is satisfactory and consistent with the state of the art for printed circuits.

The main part of the CCPS evaluation was devoted to a fatigue crack growth test on the M(T) specimen. During the FCG tests, very good stability of the readings was observed, even for the unprocessed signals from all crack gauges (Figure 7). No significant alterations in the output voltage upper envelopes were noticed after the test was initiated. A strong horizontal trend was visible between the periods of CCPS sensing layer strand breakage. Throughout the fatigue test of up to 1.5 million cycles, all CCPS sensors remained fully functional, with no failure-related changes in output signal patterns (Figure 8a). This is significant, as a resistance drift due to repeated mechanical loading is reported frequently in the literature [47,48,60]. Factors that influence this issue are mechanical (strain level, duration of applied force) as well as material (creep of the insulating layer, shape, and metallic particles content) [61].

In virtually every case, the CCPSs manifested a crack length increase in the host structure, as expected. Clear pseudo-exponential transition states were visible, which indicates a gradual tearing of the sensor’s conductive strand with crack propagation in the specimen. After a sensor’s strand was fully broken, the sensor signal reached its local maximum and stabilizes at this new level. The flat sections in the graphs (Figure 9a,b) confirm this. Moreover, the quantity of the signal level shifts for all sensors corresponded with the layout and number of strands within the sensing grid. This confirms the sensors’ correct operation, their firm integration with the host structure, and reliable strain transfer from the host. Appropriate selection of adhesive bonding methods as well as materials for sensor fabrication was carried out.

Proper sensor operation can also be confirmed by the temporal duration of the successive stable-state signal levels. In Table 5, the number of applied fatigue cycles is indicated for each CCPS strand full breakage. One can notice that these periods decrease monotonically from strand to strand. This corresponds with the crack length propagation rate and the increase in the stress intensity factor, as shown in Figure 9c. This is a typical behavior for FCG in host materials subjected to constant amplitude loading [57].

Additionally, for a standardized MT specimen as a host structure for CCPS, reference crack length measurement using a COD was possible, based on the compliance method. Comparison between the current readings from the CCPS sensors in the full strand breakage cycle (Figure 10a,b) (Table 5) and the COD reference was conducted. CCPS strand locations relative to the mechanical notch were obtained from post-test microscopic measurements. Differences in crack length comparison were highly concordant throughout the FCG test. An offset ranging from 0.4 ÷ 0.6 mm for the left side and 0.85 ÷ 1.25 mm for the right side of the specimen was achieved. Detailed offset in different stages of the FCG is presented in Figure 10a. A strong linear relation between the COD and CCPS estimated crack length was observed for both sides of the host specimen. This confirms the high consistency of the crack size increase, although with a stable shift over a crack propagation distance of almost 30 mm (Figure 10b). The FCG stop condition was set to 30 mm (based on COD measurements), which allowed for all the sensors’ strands to be fully torn.

Discrepancies between the COD and CCPS indications were analyzed to understand this issue, as well as various offset levels for the left and right sides of the specimen. Based on microscopic imaging, the results of the true crack length on the specimen breakthrough surfaces are delivered in Figure 12. The measurements revealed that the true crack length in the specimen was shorter on the left side (29.74 mm) than on the right side (30.69 mm). Additionally, there were through-thickness differences in damage size, reaching approximately 0.5 mm. This issue is frequently reported in material fatigue testing [57] and is called crack tunneling. It is commonly observed in crack growth experiments on specimens made of ductile materials such as steel and aluminum alloys. It creates a thumbnail or tunneled profile due to higher stress triaxiality and void coalescence in the center of the structure cross-section [62,63]. A higher stress intensity factor (K) can occur in the center of the specimen, resulting in a higher crack growth rate in this region. Therefore, although the crack front line is initially straight, it may later become curved, as indicated in Figure 12. Certain alloy structural orientations can also influence the development of oblique cracks. The crack tunneling effect influences the crack length values obtained using the compliance method and, consequently, the overall results of the fatigue crack propagation test. So, this is a probable reason that partially explains the offset between the COD and CCPS crack length readings. The true damage size variations between the sides of the M(T) specimen corresponded with the CCPS offset level (shorter crack on the left and lower CCPS offset compared to the right side with a larger offset). Moreover, camera observations were taken periodically during the test (Figure 13). The images confirm that the CCPS sensing layer was torn concurrently with the host structure (Figure 13c).

The crack edge post-test microscope images reveal that both the host structure and CCPS damage lines were smooth and even in the area of the inner and middle CCPS sensors. In the area of the bonded outer sensors, changes in crack line and shape were noticeable, which corresponded to a higher stress intensity factor value. Smooth and evenly spaced edges of the CCPSs and the crack line prove that sensors were properly integrated with the host. The plasma treatment was used only on the left side of the specimen and the CCPS contact surfaces. Based on the obtained results, the effect of the plasma treatment cannot be clearly determined. However, the slight visible delamination of the right-side outer sensor may indicate better adhesion strength on the plasma-treated side. This surface engineering method will be investigated further in the future.

Furthermore, the manuscript introduction states that the CCPSs address specific shortcomings of some other crack gauge configurations, especially those in the form of continuous films [24,25,26,27]. CCPS materials were chosen to enable visual observation of crack tips. Firstly, this is a challenge in the case of a non-translucent conductive or bottom layer. In [24], crack length evaluation was performed on the opposite side of a specimen using digital image correlation combined with post-test fracture surface optical inspection. It was stated that the sensor detected the presence of the crack slightly before the crack tip reached the sensor. In [25], a limited direct comparison was made between the actual crack length in a sample and sensor readings. Additionally, during both tests with the use of carbon film sensors, the resistance change with the increase in crack length was non-linear in the early stage of the sensor’s response. The accuracy of the crack length determination by the sensor was not quantified for intermediate FCG phases, as was the case in this work. In [26], in accordance with the methodology adopted by the authors for interpreting the sensor results, cracks above 27 mm could be detected. The sensor was installed in proximity to the specimen notch, so the sensor’s response time was delayed. The CCPS design allows for the verification of crack presence between grid strands, which is an additional pro-reliability feature compared to other methods.

Secondly, the CCPS grid sensor layout, composed of strands instead of a continuous film, reduces the need for data interpretation and the assessment of crack lengths. The step-wise characteristic of the sensor should be less sensitive to external disturbances, e.g., temperature and mechanical load alterations. Non-linear signal change for the foil sensing layer may lead to calibration difficulties and transition from the measured signal to the crack length. In the case of the SHM method proposed in this work, the compromise in terms of the resolution of the crack length measurement may be seen as a drawback. However, it is compensated for by the unambiguous readings, even for unprocessed signals, and the ability to customize the sensing grid layout without extra effort.

Finally, the fatigue test confirmed the CCPS resistance repeatability. The following dataset, presented in Figure 9a,b, allows for the definition of benchmark characteristics for a certain type of CCPS within a tolerance better than 10%. This demonstrates a stable and repeatable manufacturing process for sensors and individual strands. The DIW method is also found to be effective in terms of sensing layer shape customization, as the middle sensors, marked as 40019:ch2 and 4874:ch2, are characterized by a nearly 50% shorter grid. Moreover, with the DIW manufacturing method, changes in crack length measurement resolution with CCPS (defined as grid strand separation) can be rapidly introduced. If only the strand separation is modified with the same strand length and number, the final output characteristic of the CCPS will not be changed.

## 5. Conclusions

The scope of this paper is devoted to the evaluation of a new printed crack gauge, called CCPS. The materials are fabricated with an additive manufacturing method with tape casting of the bottom insulating layer, on which a conductive sensing layer and top dielectric protection are selectively deposited by the Direct Ink Write technique. The presented technique expands the range of damage detection methods for SHM, where sensors/transducers and data acquisition become an integrated part of the technical object. Compared to commercially available sensors of this type, these can be tailored in shape to fit various structural geometries. This can ensure better integration and coverage with a host component by utilizing additive manufacturing.

Within the investigation, we demonstrated that CCPSs can successfully detect and quantify the propagation of fatigue cracks in the metallic host structure. The obtained accuracy was approximately 1 mm compared to the reference COD. The offset was almost constant throughout the FCG test until a 30 mm crack size on each side of the specimen was achieved. An additional post-test microscopic crack surface analysis was also conducted. Furthermore, the sensor fabrication repeatability was verified, and preliminary results indicate that 5% tolerance in the output resistance characteristic can be achieved for a batch of production. The DIW method is found to be effective in sensing layer shape customization. The sensing grid layout can be rapidly adapted to the host structure by means of size and crack length measurement resolution.

Further research on CCPS will focus on performance and stability verification under external factors. Temperature/humidity aging and post-aging fatigue crack monitoring capabilities will be evaluated.

## Figures and Tables

**Table 1:** M(T) specimen material properties [54].

Al 2024-T3			
*R_m_* (MPa)	*R_p_*_0.2_ (MPa)	*E* (MPa)	*ν* (—)
483	345	73,100	0.33

**Table 2:** Specimen geometrical parameters for compliance method.

Parameter Name	Symbol	Value
Specimen thickness	B	3 mm
Specimen width	W	100 mm
Specimen overall length	H	264 mm
Mechanical notch length	a_n_	10 mm
Current crack length	a	Test variable in mm
Applied force amplitude	P	Test set parameter in kN

**Table 3:** CCPS sensors’ resistance changes due to grid cutting.

No. of Connected Strands	CCPS P1 (Ω)	CCPS B1 (Ω)	CCPS B2 (Ω)	CCPS B3 (Ω)	CCPS B4 (Ω)
6	0.301	0.339	0.324	0.343	0.386
5	0.372	0.406	0.393	0.414	0.463
4	0.469	0.503	0.487	0.522	0.578
3	0.63	0.675	0.644	0.696	0.793
2	0.95	1.016	0.961	1.031	1.209
1	1.895	2.024	1.954	2.028	2.404
Short	0.244	0.24	0.242	0.242	0.211

**Table 4:** Theoretical CCPS sensors resistance characteristics based on Equations (1) and (2).

No. of Connected Strands	CCPS P1 (Ω)		CCPS B1 (Ω)		CCPS B2 (Ω)		CCPS B3 (Ω)		CCPS B4 (Ω)	
	Equation (2)	Equation (1)	Equation (2)	Equation (1)	Equation (2)	Equation (1)	Equation (2)	Equation (1)	Equation (2)	Equation (1)
6	0.316	0.301	0.337	0.339	0.326	0.324	0.338	0.343	0.401	0.386
5	0.379	0.361	0.405	0.407	0.391	0.389	0.406	0.412	0.481	0.463
4	0.474	0.452	0.506	0.509	0.489	0.486	0.507	0.515	0.601	0.579
3	0.632	0.602	0.675	0.678	0.651	0.648	0.676	0.686	0.801	0.772
2	0.948	0.903	1.012	1.017	0.977	0.972	1.014	1.029	1.202	1.158
1	1.895	1.806	2.024	2.034	1.954	1.944	2.028	2.058	2.404	2.316

**Table 5:** Specimen crack size comparison from CCPS sensors and COD.

**Crack Length by 40019:ch3 (mm)**	**Crack Length by COD (mm)**	**Delta**	**Cycle**	**Crack Length by 4874:ch1 (mm)**	**Crack Length by COD (mm)**	**Delta**	**Cycle**
1.53	N/D	N/D	pre-crack	1.74	N/D	N/D	at pre-crack
3.02	N/D	N/D	pre-crack	3.23	4.40	−1.17	219,193
4.53	4.93	−0.40	327,016	4.73	5.70	−0.97	479,955
6.06	6.43	−0.37	606,721	6.30	7.19	−0.89	736,378
7.59	7.93	−0.34	854,170	7.81	8.66	−0.85	949,648
9.09	9.60	−0.51	1,048,236	9.30	10.39	−1.09	1,113,871
**Crack length by 40019:ch2 (mm)**	**Crack length by COD (mm)**	**Delta**	**Cycle**	**Crack length by 4874:ch2 (mm)**	**Crack length by COD (mm)**	**Delta**	**Cycle**
10.42	11.03	−0.61	1,164,172	11.00	12.10	−1.10	1,233,848
11.94	12.38	−0.44	1,249,157	12.54	13.55	−1.01	1,307,755
13.47	13.83	−0.36	1,319,174	14.06	15.09	−1.03	1,361,752
14.98	15.44	−0.46	1,371,866	15.59	16.66	−1.07	1,407,813
16.51	16.93	−0.42	1,414,796	17.08	18.20	−1.12	1,444,745
18.03	18.46	−0.43	1,451,591	18.60	19.57	−0.97	1,472,772
**Crack length by 40019:ch1 (mm)**	**Crack length by COD (mm)**	**Delta**	**Cycle**	**Crack length by 4874:ch3 (mm)**	**Crack length by COD (mm)**	**Delta**	**Cycle**
20.25	20.87	−0.62	1,495,338	20.56	21.81	−1.25	1,509,966
21.82	22.31	−0.49	1,517,938	22.08	23.03	−0.95	1,528,427
23.27	23.61	−0.34	1,535,092	23.57	24.80	−1.23	1,548,319
24.84	25.43	−0.59	1,554,162	25.11	26.16	−1.05	1,560,875
26.28	26.59	−0.31	1,564,355	26.57	27.75	−1.18	1,571,765
27.86	28.09	−0.23	1,573,672	28.10	29.26	−1.16	1,579,791

**Table 6:** Statistics of the CCPS resistance measurements for tested sensors.

No. of Connected Strands	Minimum Resistance	Maximum Resistance	Mean Resistance	Standard Deviation	CV	CV (CCPS B4 Excluded)
6	0.301	0.386	0.339	0.028	8.24	5.04
5	0.372	0.463	0.410	0.030	7.38	4.01
4	0.469	0.578	0.512	0.037	7.32	3.95
3	0.630	0.793	0.688	0.057	8.37	3.91
2	0.950	1.209	1.033	0.093	9.01	3.50
1	1.895	2.404	2.061	0.178	8.65	2.78

## Data Availability

The original contributions presented in this study are included in the article. Further inquiries can be directed to the corresponding author.

## Abbreviations

The following abbreviations are used in this manuscript:

CA	Constant Amplitude
CCSP	Customized Crack Propagation Sensor
COD	Crack Opening Displacement
CPRF	Carbon-Fiber-Reinforced Polymers
CTE	Coefficient of Thermal Expansion
CV	Coefficient of Variation
DIW	Direct Ink Write
EIT	Electrical Impedance Tomography
FCG	Fatigue Crack Growth
ICM	Intelligent Coating Monitoring
LCA	Life Cycle Assessment
LED	Light Emitting Diode
LEFM	Linear–Elastic Fracture Mechanics
NDT	Non-Destructive Testing
PVD	Physical Vapor Deposition
SEC	Soft Elastomeric Capacitor
SHM	Structural Health Monitoring
